# A Novel Scoring System for Predicting the Metastases of Posterior Right Recurrent Laryngeal Nerve Lymph Node Involvement in Patients With Papillary Thyroid Carcinoma by Preoperative Ultrasound

**DOI:** 10.3389/fendo.2021.738138

**Published:** 2021-08-31

**Authors:** Kai-Ning Lu, Yu Zhang, Jia-Yang Da, Tian-han Zhou, Ling-Qian Zhao, You Peng, Gang Pan, Jing-Jing Shi, Li Zhou, Ye-Qin Ni, Ding-Cun Luo

**Affiliations:** ^1^Department of Oncological Surgery, Affiliated Hangzhou First People’s Hospital, Zhejiang University School of Medicine, Hangzhou, China; ^2^Department of Dermatology, Affiliated Hangzhou First People’s Hospital, Zhejiang University School of Medicine, Hangzhou, China; ^3^Zhejiang Chinese Medical University Affiliated Hangzhou First Hospital, Hangzhou First People’s Hospital, Hangzhou, China

**Keywords:** lymph node metastasis (LNM), papillary thyroid carcinoma (PTC), ultrasonic feature, LN-prRLN, predictive model

## Abstract

**Objective:**

Our goal was to investigate the correlation between papillary thyroid carcinoma (PTC) characteristics on ultrasonography and metastases of lymph nodes posterior to the right recurrent laryngeal nerve (LN-prRLN). There is still no good method for clinicians to judge whether a patient needs LN-prRLN resection before surgery, and we also wanted to establish a new scoring system to determine whether patients with papillary thyroid carcinoma require LN-prRLN resection before surgery.

**Patients and Methods:**

There were 482 patients with right or bilateral PTC who underwent thyroid gland resection from December 2015 to December 2017 recruited as study subjects. The relationship between the PTC characteristics on ultrasonography and the metastases of LN-prRLN was analyzed by univariate and logistic regression analyses. Based on the risk factors identified in univariate and logistic regression analysis, a nomogram-based LN-prRLN prediction model was established.

**Result:**

LN-prRLN were removed from all patients, of which 79 had LN-prRLN metastasis, with a metastasis rate of 16.39%. Multivariate logistic regression analysis revealed that LN-prRLN metastasis was closely related to sex, age, blood supply, larger tumors (> 1 cm) and capsular invasion. A risk prediction model has been established and fully verified. The calibration curve used to evaluate the nomogram shows that the consistency index was 0.75 ± 0.065.

**Conclusion:**

Preoperative clinical data, such as sex, age, abundant blood supply, larger tumor (> 1 cm) and capsular invasion, are positively correlated with LN-prRLN metastasis. Our scoring system can help surgeons non-invasively determine which patients should undergo LN-prRLN resection before surgery. We recommend that LN-prRLN resection should be performed when the score is above 103.1.

## Introduction

Thyroid cancer is the fastest growing solid tumor in the world. According to the latest global cancer report, the number of newly diagnosed thyroid cancer patients in 2020 was as high as 586,000, accounting for 11/36 (1) newly diagnosed cancers; thus, it has become a health problem of widespread public concern in recent years. At present, surgery is the first choice for papillary thyroid carcinoma (PTC) treatment, but the management of cervical lymph nodes is still controversial. It has been reported that the metastasis rate of lymph nodes posterior to the right recurrent laryngeal nerve (LN-prRLN) in patients with PTC is as high as 9.36%–31.60% (2, 3). If patients with positive LN-prRLN metastasis are not treated in a timely manner, thyroid cancer may recur, which will require the patient to undergo another operation. The difficulty and risk of multiple operations will be greatly increased, and the patient may even lose the chance of radical cure. Therefore, some researchers recommend that preventive central lymph node and LN-prRLN clearing should be performed under the premise of technical guarantees (4, 5). However, LN-prRLN resection increases the risk of injury to the right recurrent laryngeal nerve and parathyroid gland (6).

The 2015 American Thyroid Association guidelines point out that preventive central area sweeping increases the risk of temporary hypocalcemia. A meta-analysis of 23 studies conducted by Chen pointed out that preventive central area lymph node dissection will significantly increase the incidence of temporary and permanent hypoparathyroidism (7, 8). Much controversy remains regarding whether LN-prRLN resection should be performed routinely. Moreover, there are no effective methods for clinicians to identify the presence or absence of LN-prRLN metastasis. High-resolution sonography and color Doppler flow imaging are the imaging methods recommended by domestic and foreign guidelines for the diagnosis of thyroid nodules. However, because LN-prRLN are located in a deep position—surrounded by air-containing trachea, the esophagus, and cervical vertebrae—and because metastases in LN-prRLN are generally not large (some are even microscopic), they are difficult to identify by ultrasound (9). Ultrasound is very sensitive in the evaluation of primary PTC lesions, and clinicians can obtain PTC ultrasound features preoperatively (7). It has been reported that the ultrasound signs of PTC can be used to predict cervical lymph node metastasis in patients with PTC and provide a basis for the selection of surgical methods (10), but no similar study has been conducted in LN-prRLN. Therefore, this study aims to provide a theoretical basis for the preoperative clinical evaluation of LN-prRLN metastases by assessing the preoperative ultrasound features of PTC and postoperative pathological data in 482 PTC patients and analyzing the relationship between them to establish a new scoring system for LN-prRLN in patients with PTC.

## Materials and Methods

### General Information

We collected the clinical images and pathological data of 482 patients (99 men and 383 women; age range, 8–74 y, mean age 45.24 ± 11.85 y) with right or bilateral PTC treated at the Department of Oncology, Hangzhou First People’s Hospital, Zhejiang University Medical College, from December 2015 to December 2017. All 482 PTC patients underwent LN-prRLN resection. The patient inclusion criteria were as follows: 1) PTC in the right lobe or in both lobes of the thyroid confirmed by pathology after surgery, 2) the patient’s preoperative ultrasound data were complete and could be recalled, and 3) the patient underwent LN-prRLN resection. The patient exclusion criteria were as follows: 1) patients who experienced relapse or had multiple surgeries after the first treatment, 2) patients with a history of other malignant tumors, 3) patients who underwent invasive procedures, such as fine-needle aspiration or radiofrequency ablation prior to ultrasound evaluation of the lesion, and 4) patients with PTC in the isthmus or left lobe of the thyroid gland. All patients in the study signed an informed consent form.

### Ultrasound Equipment and Evaluation Methods

**Figure 1:**
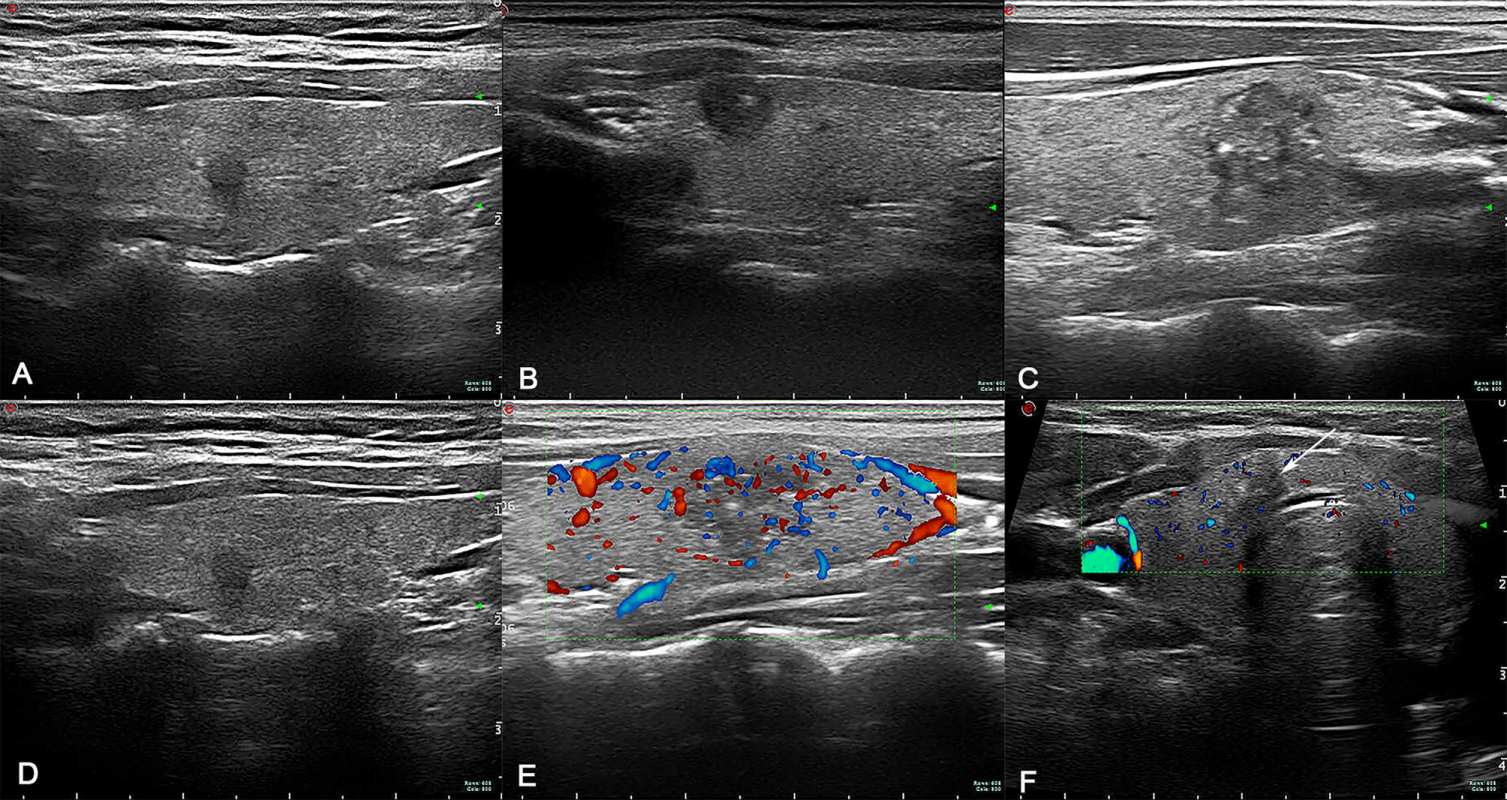
**(A)** The cancer focus is in the middle. **(B)** The cancer focus is near the anterior capsule. **(C)** Lesion with calcification. **(D)** Lesion without calcification. **(E)** Lesion with abundant blood supply. **(F)** Lesion with insufficient blood supply (arrow).

The Mylab 70 XVG and My Lab Twice Color Doppler ultrasound systems were used as the diagnostic apparatus, with a probe frequency of 7 to 13 MHz, and the instrument settings were suitable for thyroid examination. The patients were placed in a supine position, fully exposing the neck. After the suspicious malignant nodule was observed, the two-dimensional ultrasound image features of the nodule were interpreted and recorded, including the location of the lesion (upper/middle/lower/diffuse), the number of lesions (single/multiple), the maximum tumor diameter (≥ 1 cm/< 1 cm), the echo intensity (low/middle/high, cystic/solid), the echo distribution (uniform/uneven), capsular invasion (no/cling/involvement/breakthrough), edge (regular/irregular), calcification (no/yes), aspect ratio imbalance (yes/no), abundant blood supply (yes/no), and the location of the cancer focus in the sagittal plane of the thyroid gland (near the anterior capsule/middle or near the posterior capsule or near the anterior and posterior capsules). All ultrasound images in this study were retained by two physicians with higher seniority in the same team and reviewed by the same chief sonographer. The ultrasonography manifestations of PTC are presented in **Figure 1**.

### Surgical Methods

All the operations in this study were performed by thyroid specialists in cooperation with the same surgeon. The operation mode was determined according to domestic and foreign guidelines (5, 7). Right or bilateral central lymph node resection was performed at the same time as the primary focal gland lobe plus isthmus or bilateral adenectomy. The lymph node resection range included perithyroidal lymph nodes, pretracheal lymph nodes, prelaryngeal lymph nodes (Delphian lymph nodes), and tracheoesophageal groove lymph nodes, and the right side also included LN-prRLN. The upper border of LN-prRLN resection was the laryngeal entry point of the RLN, the inferior border was the intersection of the RLN with the innominate artery, the medial border was the esophagus, and the lateral boundary was the inner edge of the common carotid artery. The deep surface of LN-prRLN is the prevertebral fascia (11). Selective resection of lymph nodes in the lateral neck was performed only when there was clear evidence of metastasis.

### Statistical Analysis

In this study, we used the SPSS 26.0 package (IBM, Armonk, NY, USA) and R software (v.3.3.3) for statistical analysis. Continuous variables are expressed as the mean with standard deviation (SD) or percentage. The *t*- test was used to compare continuous variables. With the χ^2^-test or Fisher’s exact test, the relationship between the preoperative ultrasound features of PTC and LN-prRLN metastasis was analyzed. Logistic regression analysis was used to determine the risk factors for LN-prRLN metastasis. Receiver operating characteristic (ROC) curves were used to establish prediction equations and analyze the reliability of the multivariate analysis results. The risk model was established based on the risk factors determined by multiple logistic regression analysis. The consistency index (C-index) and calibration curve were calculated to evaluate the differentiation and accuracy. The level of statistical significance was set at *P* < 0.05.

## Results

### Results of LN-prRLN Resection in PTC Patients

A total of 482 patients with PTC who underwent initial surgery were included in this study, of which 79 patients had LN-prRLN metastasis, a metastasis rate of 16.39%. The number of dissected LN-prRLNs ranged from 0 to 20, with an average of 2.99 ± 2.14; the number of invaded LN-prRLNs ranged from 0 to 6.

### Single-Factor Analysis of Preoperative Ultrasound Features of PTC and LN-prRLN Metastasis

As shown in **Table 1**, the patient’s clinical characteristics such as gender, age and the preoperative ultrasound features of PTC, such as lesion location, echo pattern, tumor size, capsular invasion, calcification, blood supply, and sagittal position of thyroid cancer, were related to LN-prRLN metastasis (all *P* < 0.05). In contrast, number of cancer lesions, echo intensity, whether the edges are well defined, and whether the aspect ratio is imbalanced were not associated with LN-prRLN metastasis (all *P* > 0.05).

**Table 1:** Single-factor analysis of preoperative ultrasound features of PTC and LN-prRLN metastasis.

Risk factors		N	Metastasis of LN-prRLN		Transfer rate	p
			Yes	No		
Sex	Male		26	73	35.62%	0.003
	Female		53	330	16.06%	
Age			42.33 ± 14.18	45.81 ± 11.27		0.042
Lesion location	Upper	136	17	119	12.50%	
	Middle	183	38	145	20.77%	<0.001
	Lower	157	19	138	12.10%	
	Diffuse	6	5	1	83.33%	
Number of lesions	Single	416	66	350	15.87%	0.474
	Multiple	66	13	53	19.70%	
Echo distribution	Uniform	400	54	346	13.50%	<0.001
	Uneven	82	25	57	30.49%	
Echo intensity	Low	431	68	363	15.78%	0.253
	Middle	46	9	37	19.57%	
	High	1	0	1	0.00%	
	Cystic solid	4	2	2	50.00%	
Maximum tumor diameter	≤1 cm	366	35	331	9.56%	<0.001
	>1 cm	116	44	72	37.93%	
	No	197	20	177	10.15%	<0.001
Capsular invasion	Cling/ Involvement	242	39	203	16.12%	
	Breakthrough	43	20	23	46.51%	
	Well defined	11	2	9	18.18%	1
Margin	Poorly defined	471	77	394	16.35%	
Calcification	No	297	32	265	10.77%	<0.001
	Yes	185	47	138	34.06%	
Aspect ratio imbalance	Yes	479	79	400	16.49%	1
	No	3	0	3	0.00%	
Abundant blood supply	Yes	43	13	30	30.23%	0.016
	No	439	66	373	15.03%	
	Near anterior capsule	144	18	126	12.5%	0.002
	Middle	103	10	93	9.71%	
Location of cancer focus in the sagittal plane of the thyroid gland	Near posterior capsule	172	32	140	18.60%	
	Near anterior and posterior capsules	63	19	44	30.16%	

Location: the thyroid gland is divided into three parts in the coronal plane, and the center of the lesion near the upper pole is defined as the upper pole. The middle pole and the lower pole are defined in the same way as the upper pole. The lesion is described as diffuse when it is diffused in the thyroid.

Capsular invasion: no contact with the thyroid capsule is defined as no; contact with the thyroid membrane but the membrane is still continuous defined as cling/involvement. Aspect ratio imbalance: A/T>1. Abundant blood supply: Adler grading method; those categorized as level III have a rich blood supply. Location of cancer in the sagittal plane of the thyroid. The thyroid is divided into three parts on the sagittal plane. When the anterior and posterior capsule of the cancer focus is in the middle, it is defined as being in the middle. When one capsule of the cancer exceeds the anterior/posterior boundary, it is defined as being near the anterior/posterior capsule. When the anterior and posterior capsule of the cancer exceeds the boundary, it is defined as being near the anterior/posterior capsule according to which capsule is closer to the thyroid capsule. When the anterior and posterior capsule of the cancer is close to the anterior and posterior capsule of the thyroid gland, it is defined as the near the anterior and posterior capsule.

### Logistic Regression Analysis of Preoperative Ultrasound Features of PTC and LN-prRLN Metastasis

The two clinical characteristics and seven ultrasound lesion features with statistical significance in the single-factor analysis were included in the multivariate logistic regression analysis and assigned as follows: sex (0 for male, 1 for female), age as a continuous variable, location of the lesion (0 for upper, 1 for middle, 2 for lower, 3 for diffuse), maximum tumor diameter (0 for ≤ 1 cm, 1 for >1 cm), echo distribution (0 for uniform, 1 for uneven), capsular invasion (0 for no, 1 for cling involvement, 2 for breakthrough), calcification (0 for no, 1 for yes), abundant blood supply (0 for no,1 for yes), and location of the cancer focus in the sagittal plane of the thyroid gland (0 for near the anterior capsule, 1 for middle, 2 for near the posterior capsule, 3 for near the anterior and posterior capsules). Taking LN-prRLN metastasis as the dependent variable, the nine preoperative factors with statistical significance as independent variables were entered into the regression model for multivariate logistic regression analysis. The results showed that sex, age, and preoperative ultrasound features of PTC, such as largest diameter of the tumor, capsular invasion, and abundant blood supply, were high-risk factors for LN-prRLN metastasis (see **Table 2** for details).

**Table 2:** Logistic regression analysis of preoperative ultrasound features of PTC and LN-prRLN metastasis.

	B	SE	Wals	Sig.	Exp (B)	EXP (B) 95% CI	
						Lower limit	Upper limit
Sex	-0.684	0.299	5.242	0.022	0.504	0.281	0.906
Age	-0.023	0.011	4.338	0.037	0.977	0.956	0.999
Maximum tumor diameter	1.454	0.292	24.787	0	4.279	2.414	7.584
Capsular invasion	0.523	0.227	5.311	0.021	1.687	1.081	2.633
Abundant blood supply	0.729	0.398	3.347	0.067	2.072	0.949	4.522
Constant	-0.417	0.714	0.341	0.559	0.659		

B, Beta coefficient; SE, Standard error of the mean; Sig, Statistical significance; CI, Confidence interval.

### Logistic Regression Model to Predict the Clinical Value of Risk Factors for LN-prRLN Metastasis

**Figure 2:**
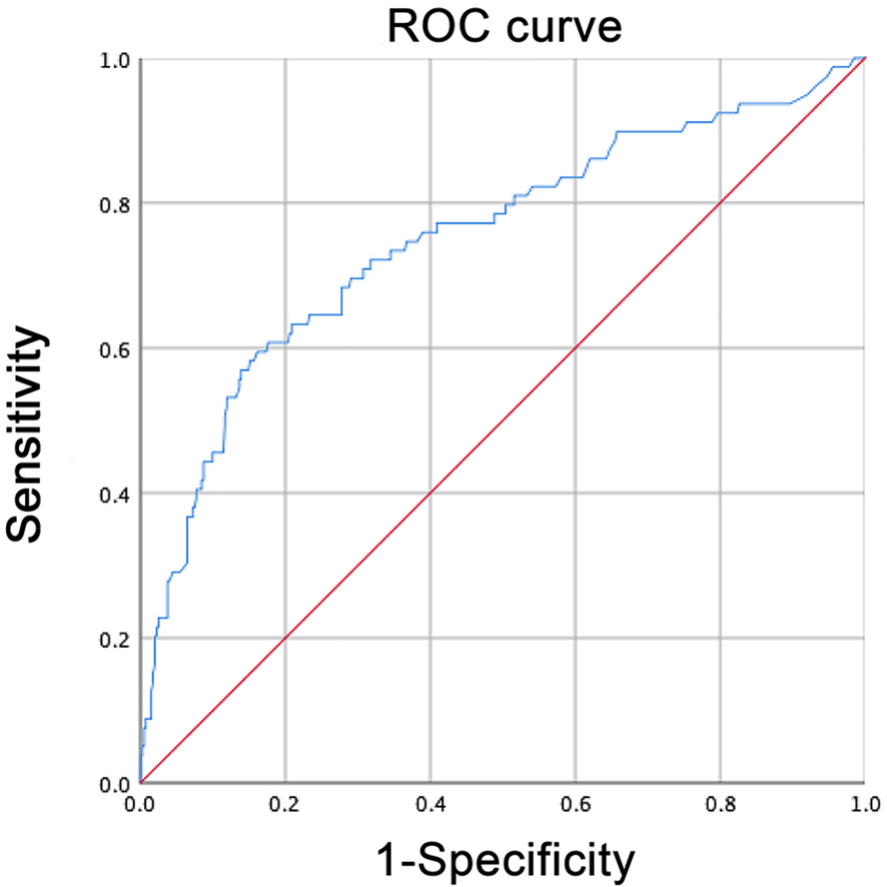
ROC curve of the logistic regression model for predicting LN-prRLN metastasis.

A logistic regression model was established based on the results of the multivariate analysis: logistic (P)= -0.417– 0.023 × age – 0.684 × sex + 1.454 × the maximum tumor diameter + 0.523 × capsular invasion+0.729 × abundant blood supply. The values of each factor were substituted into the equation to obtain the probability of multifactor joint prediction, and the ROC curve was calculated (**Figure 2**). The predictive model comprehensively reflects the predictive value of the five risk factors. The area under the curve (AUC) of the model for predicting LN-prRLN metastasis was 0.75 (95% CI, 0.685–0.815), the cut-off value was 0.235, the sensitivity was 59.5%, the specificity was 83.9%, and the maximum Youden index was 0.434.

### Nomogram of LN-prRLN and Its Verification

**Figure 3:**
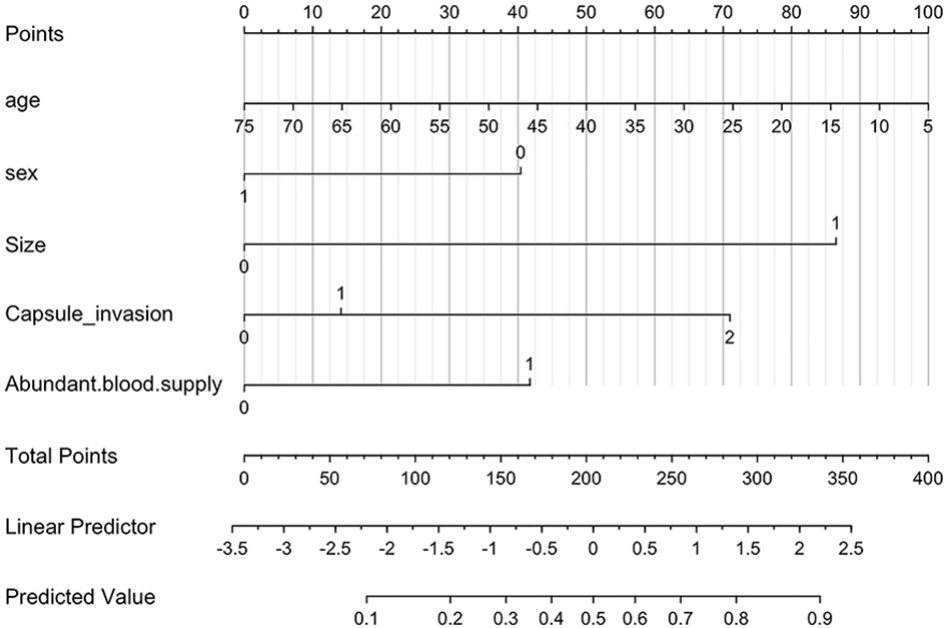
Nomogram for predicting the risk of LN-prRLN metastasis. The linear predictor is the coordinate axis of the linear prediction value, and the linear prediction value is transformed into the corresponding probability value through a conversion function.

**Figure 4:**
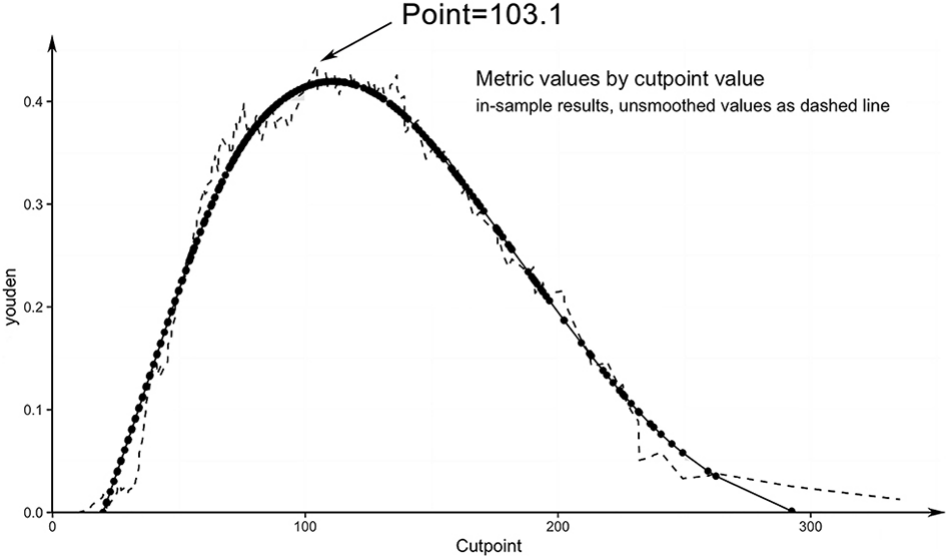
Youden Index.

All variables identified as high-risk factors in the multivariate analysis were included in the LN-prRLN nomogram (**Figure 3**). The nomogram showed that sex, age, tumor size greater than 1 cm, capsular invasion, and abundant blood flow were all associated with a higher risk of LN-prRLN metastasis. Each risk factor can be converted into a corresponding score according to the nomogram. Starting from the age of 75, the score increases by 1.43 for every year of age until the age of 5. Male patients had a score of 40.39, while capsular invasion (cling/involvement), capsular invasion, abundant blood supply, and larger tumors (>1 cm) had scores of 14.13, 70.98, 41.73, and 86.49, respectively. Physicians can add the individual scores according to the characteristics of the patient’s primary lesion to get the total score. Then clinicians can estimate the risk of LN-prRLN metastasis based on the total score (**Figure 4**) to develop individualized treatment plans for the patient.

**Figure 5:**
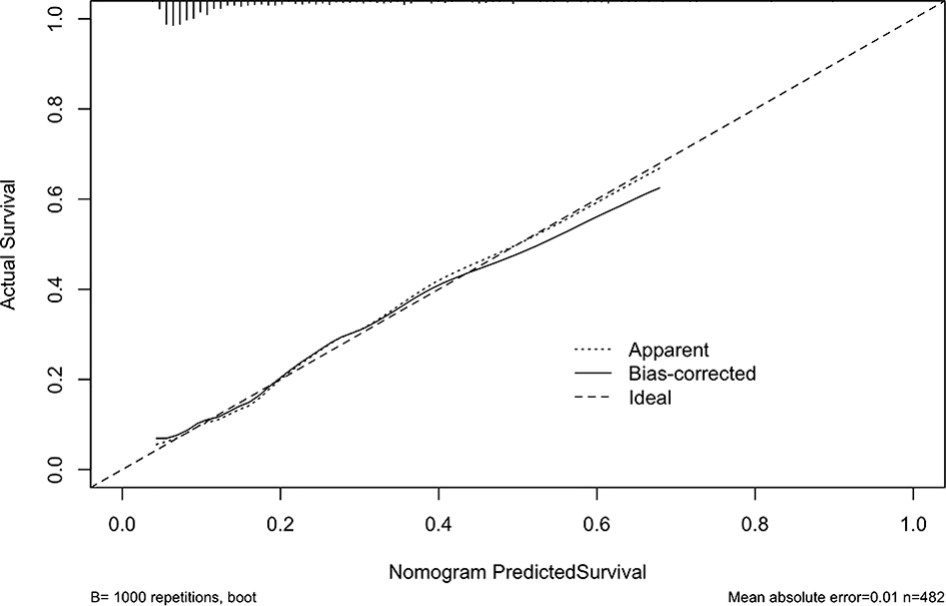
Bootstrap Method. The x-axis shows the probability of LN-prRLN metastasis predicted by the nomogram and the y-axis shows the actual ratio of LN-prRLN metastasis. The reference line is a dashed line, indicating the apparent calibration.

We drew a calibration curve to evaluate the nomogram, and the calibration curve showed good agreement between the prediction and observation rates (**Figure 5**). The distinguishing capability of the nomogram was then evaluated by using the C-index, which was 0.75 ± 0.065.

## Discussion

PTC accounts for 84% of all thyroid cancers (12), and it is the main type of new thyroid cancer cases seen in the clinic. Although the majority of PTC patients have a 3-year or 5-year survival rate of more than 90% after standard diagnosis and treatment, many patients already have cervical lymph node metastasis when they are diagnosed with PTC. The relevant literature reports that the metastasis rate of LN-prRLN ranges from 5.80% to 31.60% (2, 3, 11, 13). In this study, the metastasis rate of LN-prRLN in PTC patients was 16.39%, which was consistent with that in the above literature. Therefore, proper treatment of LN-prRLN is an important part of the surgical treatment of PTC. For the PTC patients with CN+, the Chinese expert consensus on the diagnosis and treatment of papillary thyroid microcarcinoma (2016 version) recommends that not only the traditional central lymph nodes should be cleaned but also the LN-prRLN should be cleared (5). Although domestic and foreign guidelines have not reached a consensus on PTC patients with cN0, lymph node metastases are associated with local and regional recurrence and distant metastasis, affecting disease-free survival rate. Therefore, the authors suggest that preoperative evaluation of cervical lymph nodes (including LN-prRLN) should be emphasized in patients with PTC.

Ultrasound is not sensitive to the diagnosis of metastatic LN-prRLN because of the interference from the trachea, esophagus, and surrounding tissues. Yang et al. reported that the sensitivity, specificity, and accuracy of ultrasound for cervical lymph node metastasis in PTC patients were 38%, 88%, and 57%, respectively (14). It can be speculated that the diagnostic value of ultrasound for LN-prRLN is lower. In terms of the indirect prediction of LN-prRLN metastasis, Li et al. performed a meta-analysis of 10,014 PTC patients and pointed out that the risk of LN-prRLN metastasis in PTC patients was closely related to the clinicopathological features of cancer, including tumor size, capsular invasion, and the number of lesions (15). Ting et al. also pointed out that the characteristics of PTC were related to LN-prRLN metastasis in 301 patients with PTC (16). However, the characteristics of PTC can be accurately evaluated by color Doppler ultrasound diagnostic equipment, which provides the possibility for clinicians to predict LN-prRLN metastasis through preoperative ultrasound images.

Based on univariate and multivariate logistic regression analyses, we found a significant correlation between tumor size and LN-prRLN metastasis, which was consistent with the findings of most studies (11, 15, 17). In addition, many studies have shown that extraglandular invasion increases the risk of LN-prRLN metastasis (18). In this study, univariate and multivariate logistic regression analyses also suggested that extraglandular invasion is a high-risk factor for predicting LN-prRLN metastasis. Moreover, Wendong et al. (19) demonstrated the correlation between cervical lymph node metastasis and lymphatic reflux through nanocarbon tracing technology. Zhang et al. (20) also reported that the probability of cervical lymph node metastasis is reduced when the primary focus is located on the upper pole of the thyroid gland. Wang et al. (21) found that the rate of cervical lymph node metastasis in PTC that occurred in the upper pole was lower than that in the middle and lower poles. The results of this study suggested that the closer the primary PTC was to the middle and lower poles and the closer it was to the capsule, the greater the probability of LN-prRLN metastasis. All the above results indicated that the location of PTC could predict whether cervical lymph node metastasis occurred. Chen et al. (22) suggested that there was a significant correlation between internal blood flow and cervical lymph node metastasis in PTC patients, and the more abundant the blood flow was, the greater the probability of metastasis. This study also suggests that blood flow abundance is an independent predictor of LN-prRLN metastasis, consistent with the findings of the above literature.

The area under the curve (AUC) of the logistic regression model was 0.75 (95% CI, 0.685–0.815), the cut-off value was 0.235, the sensitivity was 59.5%, the specificity was 83.9%, and the maximum of the Youden index was 0.434. Its sensitivity for predicting lymph node metastasis in patients with PTC is superior to that of traditional ultrasound or CT. When the predicted point is > 103.1, the existence of LN-prRLN metastasis should be considered. Calcification, one of the ultrasound signs, is an important feature for identifying benign and malignant tumors, and it is also used in PTC. Recent studies have shown that PTC with calcification is a predictor of cervical lymph node metastasis (23, 24). The results of this study showed that the metastasis rate of LN-prRLN in the PTC with calcification group (25.41%) was significantly higher than that in the noncalcification group (10.77%). However, in multivariate logistic regression analysis, calcification did not show a predictive advantage, so whether calcification has value in predicting LN-prRLN metastasis still needs to be further confirmed.

After determining the risk factors for lymph node metastasis, we established a scoring system, and this scoring system was proven to be reliable by using the C-index and calibration curves. Therefore, clinicians can assess the risk of LN-prRLN metastasis before surgery based on this simple scoring system. Because this scoring system can provide a theoretical basis, physicians can explain to patients why LN-prRLN resection is necessary. If the patient’s score is greater than 103.1, the clinician should note that the patient has a high risk of LN-prRLN metastasis.

This study has some limitations. First, this is a retrospective study with inherent selection bias. Second, without external verification data, the statistical power was decreased.

In summary, sex, age, and the preoperative assessment of PTC characteristics are helpful in determining the metastasis to LN-prRLN: diameter of the primary lesion greater than 1 cm, capsular invasion, and abundant blood flow are high-risk factors for LN-prRLN metastasis. We also set up a scoring system to help surgeons determine which patients need LN-prRLN resection before surgery. This scoring system can predict lymph node metastasis in patients without invasive procedures, reducing patients’ pain and promoting the rational use of medical resources. When the patient’s score exceeds 103.1 points, we recommend LN-prRLN resection.

## Data Availability Statement

The raw data supporting the conclusions of this article will be made available by the authors, without undue reservation.

## Ethics Statement

The studies involving human participants were reviewed and approved by Ethics Committee of Hangzhou First People’s Hospital. The patients/participants provided their written informed consent to participate in this study.

## Author Contributions

K-NL: conception and design, provision of study materials or patients, data analysis and interpretation, and manuscript writing. YZ: conception and design, provision of study materials or patients, data analysis and interpretation, and manuscript writing J-YD: conception and design, provision of study materials or patients, data analysis and interpretation, and manuscript writing. T-hZ: data analysis and interpretation and manuscript writing. L-QZ: data analysis and interpretation and manuscript writing. YP: provision of study materials or patients and manuscript writing. GP: provision of study materials or patients and manuscript writing. J-JS: provision of study materials or patients and manuscript writing. LZ: provision of study materials or patients and manuscript writing. Y-QN: data analysis and interpretation and manuscript writing. D-CL: conception and design, administrative support, provision of study materials or patients, and manuscript writing. All authors contributed to the article and approved the submitted version.

## Funding

This work was supported by the Major Scientific and Technological Innovation Projects of Hangzhou Province (Grant number 20131813A08), the Medical Health Science and Technology Project of Zhejiang Provincial Health Commission (Grant numbers 0020190490 and 0020190151), Medical and Health Technology Project of Hangzhou (Grant number A20200432), and the Zhejiang Provincial Medical and Health Technology Project (Grant number 2019RC240).

## Conflict of Interest

The authors declare that the research was conducted in the absence of any commercial or financial relationships that could be construed as a potential conflict of interest.

## Publisher’s Note

All claims expressed in this article are solely those of the authors and do not necessarily represent those of their affiliated organizations, or those of the publisher, the editors and the reviewers. Any product that may be evaluated in this article, or claim that may be made by its manufacturer, is not guaranteed or endorsed by the publisher.
